# Fumaric acid ester-induced renal Fanconi syndrome: evidence of mitochondrial toxicity

**DOI:** 10.1093/ckj/sfaa270

**Published:** 2021-01-11

**Authors:** Elizabeth R Wan, Keith Siew, Lauren Heptinstall, Stephen B Walsh

**Affiliations:** Department of Renal Medicine, Royal Free Hospital, University College London, London, UK; Department of Renal Medicine, Royal Free Hospital, University College London, London, UK; Department of Renal Medicine, Royal Free Hospital, University College London, London, UK; Department of Renal Medicine, Royal Free Hospital, University College London, London, UK

**Keywords:** acute kidney injury, chronic kidney disease, Fanconi syndrome, proteinuria, tubular

## Abstract

**Background:**

Fumaric acid esters (FAEs) are used to treat chronic plaque psoriasis. Fumarate is a crucial component of the Krebs cycle and mitochondrial function. Proximal tubule cells have high energy demands and rely on aerobic respiration. Proximal tubular dysfunction can cause renal Fanconi syndrome and acute kidney injury. We sought to better understand the mechanism for this in the context of FAE therapy.

**Methods:**

We describe a case series of 10 patients with FAE-associated Fanconi syndrome. Patients were diagnosed and managed at a tertiary renal tubular disorder clinic, with examination of serum and urine biochemistry. Five patients had a renal biopsy with examination of the specimens by electron microscopy.

**Results:**

The median age was 36.5 years [interquartile range (IQR) 32.25–54.25]. The median dose of FAE was 720 mg/day (IQR 390–720). There was low molecular weight proteinuria: the median urinary retinol-binding protein (RBP) at presentation was 8385 μg/mL (IQR 2793–14 600) and the RBP:creatinine ratio was 710 (IQR 390–2415). All patients had hyperphosphaturia [median fractional excretion of phosphate 24.2% (IQR 20.8–26.9), normal range <20%] as well as relative hypophosphataemia, with a median serum phosphate concentration of 0.93 mmol/L (IQR 0.83–0.97). Renal histology showed proximal tubular damage and abnormal mitochondrial morphology. Two patients had a favourable biochemical response to treatment with probenecid.

**Conclusions:**

We document for the first time that FAE-associated renal Fanconi syndrome is associated with mitochondrial damage visible on electron microscopy. This effect may be ameliorated by antagonism of the organic anion transporter with probenecid.

## INTRODUCTION

Fumaric acid esters (FAEs) are immunomodulatory agents used to treat chronic plaque psoriasis in Germany since 1994 [a], although their effect on psoriasis was described in 1959 [2]. Although they are a standard first-line psoriasis treatment in Germany [3], FAEs are a third-line treatment for severe, non-responsive plaque psoriasis in the UK [4]. The most common commercially available formulation is Fumaderm (Biogen, Germany), consisting of dimethylfumarate and three other FAEs [5]. Recently dimethylfumarate has been licensed as a treatment for relapsing–remitting multiple sclerosis [6]. While there has been some debate about the toxicity of FAEs [7], case reports and one retrospective cross-sectional study describe a risk of renal proximal tubular dysfunction [8] that may manifest as renal Fanconi syndrome [1] or as acute kidney injury (AKI) [9]. Renal Fanconi syndrome describes a pattern of reduced reabsorption of a number of solutes by the proximal convoluted tubule, causing urinary wasting of bicarbonate, phosphate, glucose, urate, amino acids and low molecular weight proteins such as retinol-binding protein (RBP). It is usually an acquired disorder, often due to drug toxicity [10]. Along with the risk of developing kidney injury, sufferers are predisposed to osteomalacia and pathological fractures due to hypophosphataemia.

The first component of the Krebs cycle to be identified was fumarate, securing the 1937 Nobel Prize for Physiology or Medicine for Albert Szent-Györgyi [11]. Fumarate is the intermediary resulting from the dehydrogenation of succinate in the Krebs cycle. Proximal tubule cells have a high adenosine triphosphate (ATP) demand due to their role in actively transporting molecules from the filtrate and are heavily dependent on mitochondrial aerobic respiration [12]. We therefore hypothesized that FAEs were likely to act as mitochondrial toxins.

Further, fumarate is a specific substrate of the organic anion transporter 1 (OAT1), a basolateral proximal tubular cell transporter [13]. Probenecid is a prototypical OAT inhibitor, blocking both OAT1 and 3 (two of the OAT solute carrier family that are found in the kidney) [14]. We therefore reasoned that inhibiting OAT with probenecid should prevent delivery of the drug to the proximal tubule cells, and thus renal toxicity. Such a strategy is already employed with some nephrotoxic therapies (e.g. cidofovir [15]).

## MATERIALS AND METHODS

### Patient population

All patients were referred to the University College London (UCL) tubular clinic for diagnosis and management. All were taking FAEs. Cases were diagnosed between 2010 and 2017. Patients were investigated with serum and urine biochemistry, analysed according to standard operating procedures in the Department of Biochemistry. Five patients had a renal biopsy, with examination of the specimens by light and electron microscopy.

### Statistical analysis

Data were analysed with descriptive statistical methods using R [16] (R Foundation for Statistical Computing, Vienna, Austria) in RStudio [17] (RStudio, Boston, MA, USA).

#### Case vignettes

*Case 1*. A 46-year-old man was referred due to proteinuria and an incremental increase in his serum creatinine (to 140 µmol/L). He had been diagnosed with chronic plaque psoriasis 20 years previously and had been treated over the years with a wide range of therapies [steroids, psoralen and ultraviolet A radiation therapy (PUVA), methotrexate, acitretin, cyclosporin, etanercept and infliximab]. He started FAEs in March 2010 at a dose of 480 mg with a good response in his skin disease. Renal Fanconi syndrome was diagnosed on the basis of a elevated urinary fractional excretion of phosphate (FE_PO4_) and low molecular weight proteinuria (LMWP) (see Supplementary data, Table S1). FAEs were stopped, but his estimated glomerular filtration rate (eGFR) did not improve. Six months later he underwent a renal biopsy that showed features of mild acute tubular damage with some background chronic damage. At the last clinic follow-up, his psoriasis was poorly controlled on topical therapy.

*Case 2*. A 32-year-old woman who had been taking FAEs for 2 years was referred after a repeated finding of blood and protein in her urine and an increase in her serum creatinine following a dose increase to 720 mg. She had a diagnosis of psoriasis and psoriatic arthritis and had previously had Stevens–Johnson syndrome after sulfasalazine. Her serum phosphate had been as low as 0.25 mmol/L (normal range 0.87–1.45). She had an RBP of 19 300 µg/L, an FE_PO4_ of 27% (normal range <20) and hypouricaemia at 82.1 µmol/L (normal range 200–420). The dose of FAEs was reduced and probenecid started to reduce proximal tubular toxicity. However, she developed a skin eruption and this was stopped. She underwent a renal biopsy; while light microscopy was unremarkable, electron microscopy showed widespread mitochondrial dysmorphology with a ‘blown-up’ appearance and disruption of the cristae. She remains on FAEs and under follow-up.

*Case 3*. A 33-year-old woman was referred with intermittent blood and protein on urinalysis. She had been prescribed 360 mg FAEs and monthly adalimumab injections for chronic plaque psoriasis; her dermatologist had measured her urinary RBP, which was elevated at 1320 µg/L. She underwent a renal biopsy and electron microscopy demonstrated enlarged mitochondria with distorted cristae. She was trialled on probenecid 500 mg twice a day and her RBP fell to a nadir of <100 µg/L. In fact, there was a sustained decrease in the RBP:creatinine ratio to 50, even when FAEs had to be increased to 600 mg/day for a psoriasis flare. Serum urate and phosphate also rose with probenecid therapy.

*Case 4*. A 57-year-old man was referred from another tertiary hospital with an abnormal but stable creatinine level (115 µmol/L). He had been taking FAEs for 12 years, to a maximum dose of 720 mg, and had experienced glycosuria and proteinuria throughout this time. A vasculitis screen, ultrasound scan of the kidney and urogenital tract and intravenous urogram at his local centre were unremarkable. He was found to have an elevated FE_PO4_ (20.7%) and urinary RBP (10 200 µg/L), with a low serum urate level (190 µmol/L). FAEs were stopped and he was switched to adalimumab, with good control of his psoriasis. Despite this, the proximal tubulopathy persisted. He declined a renal biopsy. Eventually, 2 years after stopping the drug, his serum and urine biochemistry normalized.

*Case 5*. A 70-year-old man was referred for assessment from a tertiary dermatology centre due to proteinuria. He had psoriasis for 45 years and had been on FAEs for the last 24 years. He had an elevated urinary RBP and elevated FE_PO4_ of 26%. FAEs were stopped. Unfortunately the patient developed severe pustular psoriasis requiring admission to the intensive care unit. He is now well and controlled on adalimumab.

*Case 6*. A 28-year-old woman was referred with heavy proteinuria (+++) on urinalysis and previous hypophosphataemia. At referral she had been taking 720 mg FAEs in addition to etanercept for 3 years. She had a renal biopsy that appeared normal by light microscopy, but enlarged mitochondria with disrupted cristae were demonstrated on electron microscopy. Renal Fanconi syndrome was managed by reducing the dose of FAEs to 360 mg and psoriasis control was optimized by switching etanercept to adalimumab. Renal Fanconi syndrome resolved with this approach.

*Case 7*. A 39-year-old man was referred for assessment due to urinalysis showing glycosuria, microscopic haematuria and proteinuria. He had recently increased the dose of FAEs to 720 mg/day from 600 mg/day. At clinic review his creatinine was elevated at 114 µmol/L, the FE_PO4_ was 23.2% and urinary RBP was 59 900 µg/L. He went on to have a renal biopsy and light microscopy showed features of acute tubular damage, particularly to the proximal tubules. Electron microscopy showed a ‘blown up’ appearance of the mitochondria. FAEs were stopped, with a plan to start biologics. This was delayed for 3 months while he was treated for latent tuberculosis. After this treatment he was started on adalimumab, with a good skin response.

*Case 8*. A 34-year-old man with chronic plaque psoriasis was referred to the UCL tubular clinic with a 1-year history of dipstick proteinuria and microscopic haematuria. He was taking FAEs, having previously failed acitretin and methotrexate therapy. He was started on probenecid 500 mg twice a day, which was well tolerated, and continued on the same dose of FAEs. His urinary RBP fell from 939 to 570 µg/L. He remains under clinic surveillance.

*Case 9*. A 29-year-old man was referred with an abnormal urine dip (blood and protein) and a deterioration in his serum creatinine from 70 to 91 µmol/L. He had been taking 720 mg FAEs for a year, having been unable to tolerate a lower dose due to psoriasis flares and having experienced liver toxicity from methotrexate. He was found to have an elevated urinary RBP of 13 100 µg/L, with a low-normal serum phosphate of 0.89 mmol/L. The same dose of FAEs was continued with close monitoring.

*Case 10*. An 81-year-old man was referred to the nephrology service with chronic renal impairment and glucose, blood and protein on urinalysis. He had lived for 40 years with psoriasis, at times with up to 97% skin coverage. He had been treated previously with a wide range of therapies, most recently stopping cyclosporine due to nephrotoxicity. He had been treated with FAEs for the last 9 years. He declined a renal biopsy and a diagnosis of renal Fanconi syndrome was made on serum and urinary biochemistry. He continued to take FAEs at a much-reduced dose of 60 mg once a day. He had a normal serum and urine phosphate at follow-up.

## RESULTS

We describe 10 patients, all of whom were diagnosed at our unit. Seventy percent of the cases were men; the median age at diagnosis of renal Fanconi syndrome was 36.5 years [interquartile range (IQR) 32.25–54.25]. The most frequent ethnic group was white British (50%). The median dose of FAEs was 720 mg (IQR 390–720), with a median dose by weight of 8.71 mg/kg (IQR 5.90–10.29). Biochemistry is summarized in Table 1 and individualized data are available in Supplementary data, Table S1. LMWP was present: the median urinary RBP at presentation was 8385 μg/mL (IQR 2793–14 600) and the RBP:creatinine ratio was 710 (IQR 390–2415). All patients had relative hypophosphataemia, with a median serum phosphate concentration of 0.93 mmol/L (IQR 0.83–0.97). All patients had an elevated FE_PO4_ [median 24.2% (IQR 20.8–26.9), normal range <20%]. Hypouricaemia was also prevalent [median serum urate concentration 150 μg/L (IQR 145–190)].

**Table 1.** Summary data for case series

Variable	Normal range	Median (IQR)
Age at diagnosis ( years)		36.5 (32.25–54.25)
Weight (kg)		70.58 (62.5–74.78)
Dose FAEs in 24 h (mg)		720 (390–720)
Dose FAEs by weight (mg/kg)		8.71 (5.90–10.29)
Creatinine (µmol/L)	0.66–112	81 (65–113)
eGFR (mL/min/1.73 m^2^)		98.5 (61.75–107.75)
Sodium (mmol/L)	135–145	142 (141–143.5)
Potassium (mmol/L)	3.5–5.1	4.05 (3.0–4.43)
Uric acid (µmol/L)	200–420	150 (145–190)
Inorganic phosphate ( mmol/L)	0.87–1.45	0.86 (0.80–1.01)
Urine RBP (µg/L)		8385 (2793–14 600)
Urine RBP:creatinine ratio^a^	<14	709.5 (390.25–2415.25)
Urine creatinine ( µmol/L)		8500 (4440–11 600)
Urine inorganic phosphate (mg/dL)		14.2 (11.50–20.0)
Fractional excretion phosphate (%)	<20	24.2 (20.8–26.9)

a Calculated as creatinine (µmol/L)/RBP (µg/L).

**FIGURE 1:**
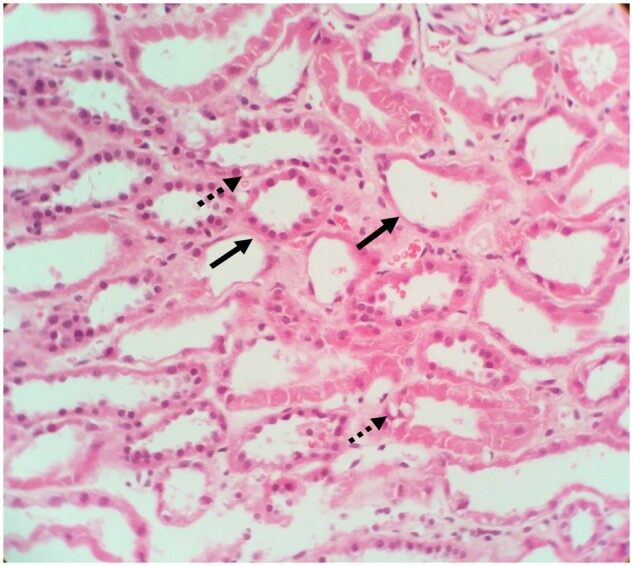
Haematoxylin and eosin staining, original magnification ×400. Tubules show acute damage indicated by irregular flattening (solid arrows) and occasional small cytoplasmic vacuolations (dashed arrow).

**FIGURE 2:**
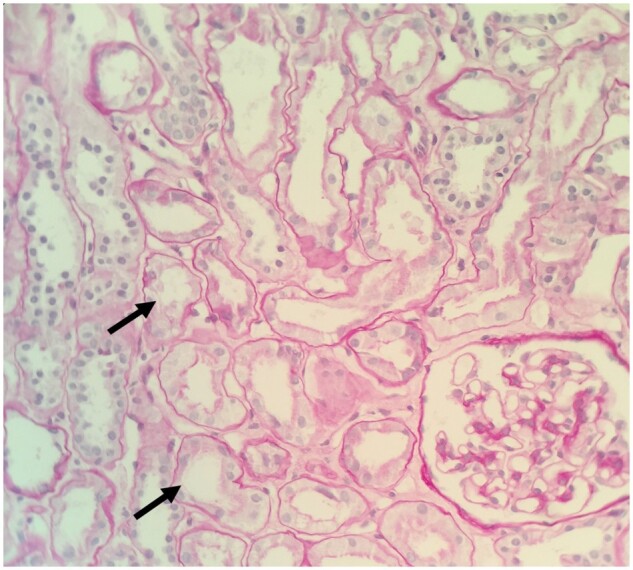
Periodic acid–Schiff staining, original magnification ×400, highlighting the patchy loss of the brush border (solid arrows).

**FIGURE 3:**
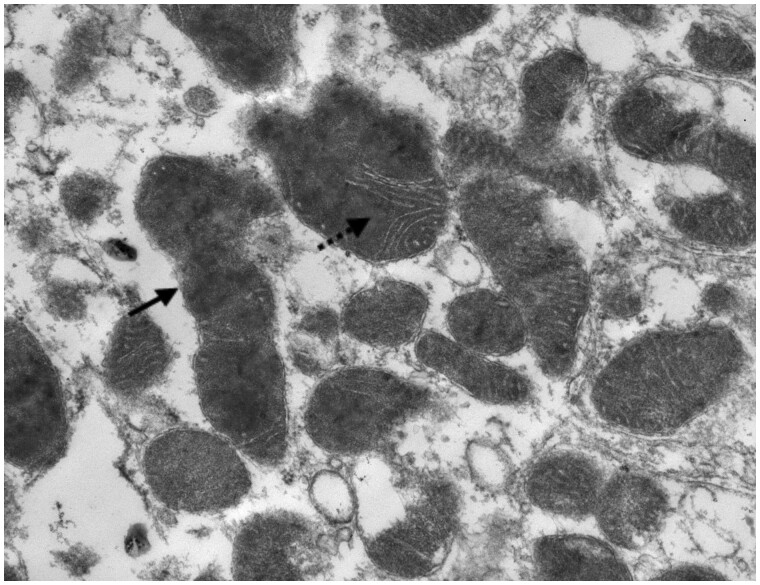
Electronic microscopy image, visualized at ×6000, showing the proximal tubules with focal mitochondrial changes, including swollen forms (solid arrow) and distorted cristae (dashed arrow).

Five patients underwent renal biopsy. In two of the five biopsies there were features of acute damage to the proximal tubules visible on light microscopy (Figure 1), with irregular flattening of the tubules, and occasional small cytoplasmic vacuolations visible on simple haemotoxylin and eosin staining. Periodic acid–Schiff staining highlighted the patchy loss of the brush border (Figure 2). Glomeruli were normal. The remaining three biopsies were superficially normal with light microscopy. However, electron microscopy revealed abnormal mitochondria, always with abnormal enlargement (‘blown up’ appearance) and distortion of the cristae (e.g. Figure 3). There did not appear to be any correlation between the severity of the biopsy appearance and the severity of the LMWP, which is generally considered the most sensitive measure of proximal tubular dysfunction in renal Fanconi syndrome.

While most patients stopped the drug or were managed at lower doses, three patients were treated with probenecid. In one of these cases the patient terminated treatment after a severe skin reaction. However, in the remaining two we saw a biochemical response, with a reduction in the urinary RBP (Table 2).

**Table 2.** Probenecid treatment

Case	Baseline urine RBP (µg/L)	Baseline RBP:creatinine ratio^a^	Urine RBP after treatment (µg/L) (% reduction)	RBP:creatinine ratio after treatment (% reduction)
4	1320	228	181 (86)	43 (81)
9	6570	939	570 (91)	31 (97)

a Calculated as creatinine (µmol/L)/RBP (µg/L).

## DISCUSSION

As proximal tubular toxins, FAEs may cause both renal Fanconi syndrome [1] and even AKI [9]. As previous authors have noted, development of Fanconi syndrome may herald AKI [18]. However, stopping FAE treatment is not without risk. FAEs are used as a third-line agent in the UK [4], so patients have resistant or severe disease. Indeed, in one of our cases, cessation of FAEs resulted in the patient developing life-threatening pustular psoriasis.

Our data support the mechanism of proximal tubular injury by FAEs being mitochondrial toxicity. This is plausible. Proximal tubule cells are involved in the active transport of many solutes to and from the tubule lumen and are metabolically very active. They possess a dense population of mitochondria for the production of ATP [19] and. as they have little glycolytic ability, they are heavily dependent on aerobic respiration [20]. Mitochondria are dynamic organelles that can undergo both fission (in an ATP-depleted environment) and fusion (e.g. damaged mitochondria might fuse to create enlarged, healthy organelles).

Malate, another Krebs cycle component, can cause renal Fanconi syndrome. In fact, malate-induced renal Fanconi syndrome in mice is a well-established model of both renal Fanconi syndrome and acute renal failure [21, 22]. Malate results from the hydration of fumarate catalysed by fumarase. Malic acid is also a specific substrate of OAT1 [13], present on proximal tubular cells. Malic acid depresses the sodium (Na)–potassium (K)–ATPase activity in the proximal convoluted tubule [23], consistent with ATP depletion. Na–K–ATPase activity in distal renal segments is unaffected by the drug [24], possibly due to reduced OAT expression there [25] or greater glycolytic capability of that nephron segment. The closely related malonic acid was recognized as early as 1972 [26] to cause morphological changes in mitochondria by disrupting the Krebs cycle. There may be a single common pathway of injury that is shared between malate-induced renal Fanconi syndrome and hypoxic–ischaemic AKI [21]. Indeed, fumarase itself may be a biomarker of AKI [27].

Therefore there may be some parallels in the mechanism of toxicity induced by maleate and FAEs. However, it has recently been demonstrated in rat kidneys that malate causes small, fragmented mitochondria [28], whereas in our study the mitochondria were observed to be enlarged. An alternative mechanism of fumarate-induced nephrotoxicity might be that of ‘fumarate overflow’, as demonstrated by Chouchani *et al.* [29] in a model of ischaemia–reperfusion injury in the heart. Here, partial reversal of the malate/aspartate shuttle results in fumarate overflow, reversal of succinate dehydrogenase and accumulation of succinate. Succinate accumulation results in reverse electron transport in respiratory complex 1 and production of toxic reactive oxygen species.

In our cohort there was a slight preponderance of men, although the number of cases is small. It has been previously asserted that FAE-associated renal Fanconi syndrome is more common in women than men [30]. It is widely reported that there are sex differences in the expression of the OATs, although OAT1 is actually expressed more in males than females, at least in rats (see the review by Sekine *et al.* [14]).

These data also suggest the potential utility of treatment with probenecid in permitting continued FAE treatment. Probenecid treatment resulted in improvement in urine biochemistry in two of the three patients treated with this strategy. Unfortunately the third developed a severe skin reaction and had to terminate the probenecid. Use of this drug can cause erythroderma [15]. For those patients who are unable or unwilling to convert from FAEs to another medication (the main alternative being biologic agents), probenecid may be considered as a rescue strategy.

## CONCLUSIONS

Here we demonstrate for the first time that human FAE-associated renal Fanconi syndrome is associated with mitochondrial damage visible on electron microscopy and we hypothesize two possible molecular mechanisms. This could be better characterized by further animal models (e.g. direct comparison with the well-established malate model) and by exploring unbiased approaches in the tissue and urine, e.g. with metabolomics.

## SUPPLEMENTARY DATA

Supplementary data are available at ckj online.

## PATIENT CONSENT

Patient consent was obtained for this article.

## Supplementary Material

sfaa270_Supplementary_Table_1
