# Supplementary material for: Fumaric acid ester-induced renal Fanconi syndrome: evidence of mitochondrial toxicity
Source: Clin Kidney J. 2021 Jan 11;14(9):2085–9. doi: 10.1093/ckj/sfaa270 (PMC8894934; doi:10.1093/ckj/sfaa270)
Supplement: sfaa270_Supplementary_Table_1 [file sfaa270_supplementary_table_1.docx]

**Supplemental Table 1: Complete series data**
